# Draft genome sequence of *Gluconobacter thailandicus* NBRC 3257

**DOI:** 10.4056/sigs.4778605

**Published:** 2014-02-01

**Authors:** Minenosuke Matsutani, Haruo Suzuki, Toshiharu Yakushi, Kazunobu Matsushita

**Affiliations:** 1 Department of Biological Chemistry, Faculty of Agriculture, Yamaguchi University, Yamaguchi 753-8515, Japan; 2 Department of Environmental Science and Engineering, Graduate School of Science and Engineering, Yamaguchi University, 1677-1 Yoshida, Yamaguchi, Japan

**Keywords:** Acetic acid bacteria, *Gluconobacter*

## Abstract

*Gluconobacter thailandicus* strain NBRC 3257, isolated from downy cherry (*Prunus tomentosa*), is a strict aerobic rod-shaped Gram-negative bacterium. Here, we report the features of this organism, together with the draft genome sequence and annotation. The draft genome sequence is composed of 107 contigs for 3,446,046 bp with 56.17% G+C content and contains 3,360 protein-coding genes and 54 RNA genes.

## Introduction

Acetic acid bacteria (AAB) are strictly aerobic *Alphaproteobacteria*. AAB are well known for their potential to incompletely oxidize a wide variety of sugars and alcohols. The genus *Gluconobacter* oxidizes a wide range of sugars, sugar alcohols, and sugar acids, and can accumulate a large amount of the corresponding oxidized products in the culture medium [1]. Thus, *Gluconobacter* strains are widely used for the industrial production of pharmaceutical intermediates, such as L-sorbose (vitamin C synthesis), 6-amino-L-sorbose (synthesis of the antidiabetic drug miglitol), and dihydroxyacetone (cosmetics for sunless tanning) [1]. Furthermore, the genera *Acetobacter* and *Gluconacetobacter* are widely used for the industrial production of vinegar because of their high ethanol oxidation ability [2].

To date, six genome sequences of *Gluconobacter* strains (*Gluconobacter oxydans* 621H, *Gluconobacter oxydans* H24, *Gluconobacter oxydans* WSH-003, *Gluconobacter thailandicus* NBRC 3255, *Gluconobacter frateurii* NBRC 101659, and *Gluconobacter frateurii* NBRC 103465) are available in the public databases [3-8]. These genomic data are useful for the experimental identification of unique proteins or estimation of the phylogenetic relationship among the related strains [9-11].

*Gluconobacter thailandicus* NBRC 3257 was isolated from downy cherry (*Prunus tomentosa*) in Japan [12], and identified based on its 16S rRNA sequence [13]. Here, we present a summary of the classification and a set of features of *G. thailandicus* NBRC 3257, together with a description of the draft genome sequencing and annotation.

## Classification and features

**Figure 1:**
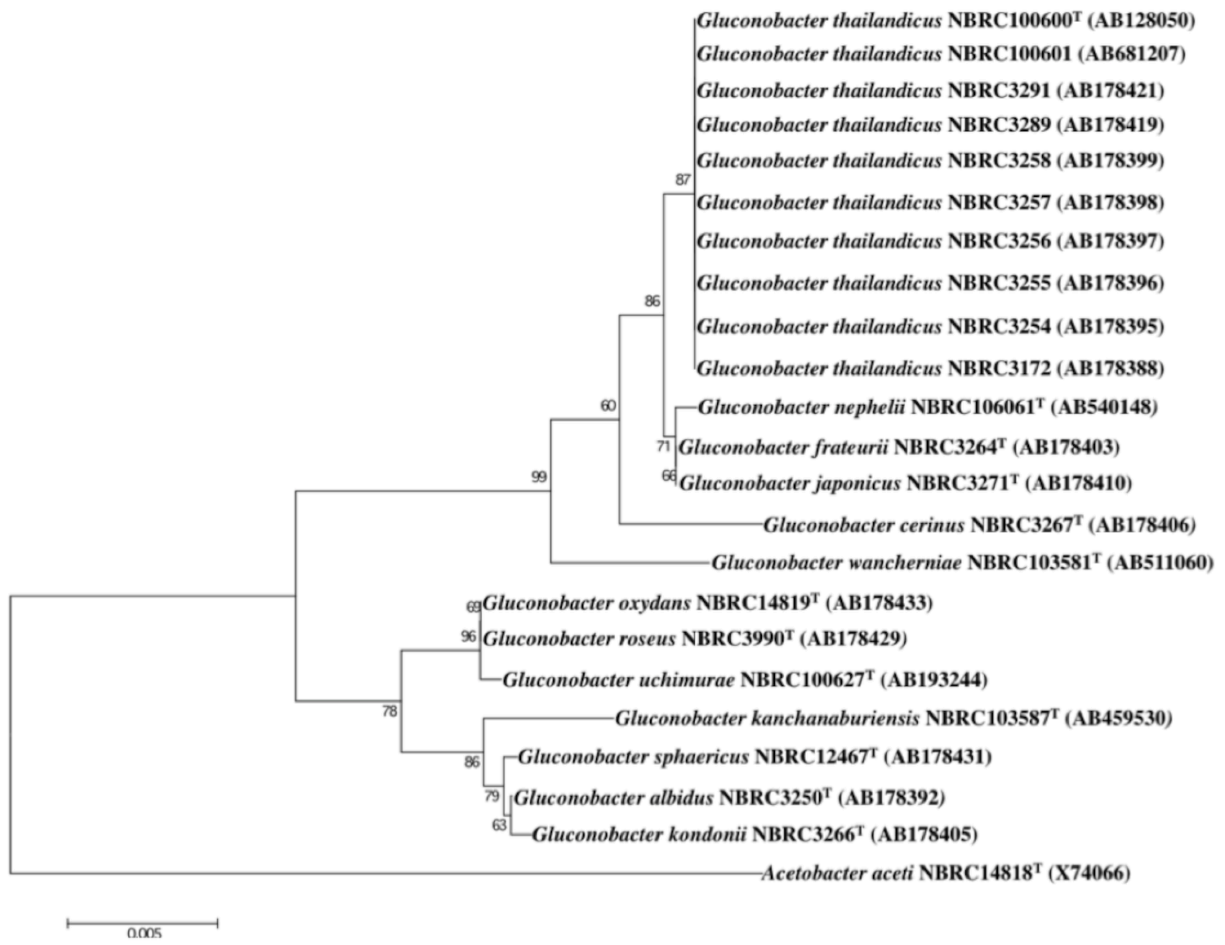
Phylogenetic tree highlighting the phylogenetic position of *Gluconobacter thailandicus* NBRC 3257 relative to other type strains within the *Gluconobacter*. To construct the phylogenetic tree, these sequences were collected and nucleotide sequence alignment was carried out using CLUSTALW [14]. We used the MEGA version 5.05 package to generate phylogenetic trees based on 16S rRNA genes with the neighbor-joining (NJ) approach and 1,000 bootstrap replicates [15,16]. *Acetobacter aceti* NBRC14818 (X74066) was used as the outgroup.

A representative genomic 16S rRNA sequence of *G. thailandicus* NBRC 3257 was compared to the 16S rRNA sequences of all known *Gluconobacter* species type strains. The 16S rRNA gene sequence identities between *G. thailandicus* NBRC 3257 and all other type strains of genus *Gluconobacter* species were 97.58-99.85%. *Gluconobacter* species (type strains) exhibiting the highest sequence identities to NBRC 3257 were *Gluconobacter frateurii* NBRC 3264^T^ and *Gluconobacter japonicas* NBRC 3271^T^. Figure 1 shows the phylogenetic relationships of *G. thailandicus* NBRC 3257 to other *Gluconobacter* species in a 16S rRNA based tree. All the type strains and ten strains of *G. thailandicus* including NBRC 3257 were used for the analysis [13,17]. Based on this tree, genus *Gluconobacter* is divided into two sub-groups. *Gluconobacter wanchamiae*, *Gluconobacter cerinus*, *G. frateurii*, *G. japonicas*, *Gluconobacter nephelli*, and *G. thailandicus* are classified as clade 1. On the other hand, *Gluconobacter kondonii, Gluconobacter sphaericus, Gluconobacter albidus, Gluconobacter kanchanaburiensis, Gluconobacter uchimurae, Gluconobacter roseus,* and *Gluconobacter oxydans* belong to the clade 2. All ten *G. thailandicus* strains are closely related to each other, and the 16S rRNA sequences have 100% identities.

Although ethanol oxidation ability is a typical feature of AAB, it is a critical feature that NBRC 3257 lacks the ability to oxidize ethanol because it is missing the cytochrome subunit of the alcohol dehydrogenase complex that functions as the primary dehydrogenase in the ethanol oxidase respiratory chain [18]. Despite its inability to oxidize ethanol, NBRC 3257 can efficiently oxidize many unique sugars and sugar alcohols, such as pentitols, D-sorbitol, D-mannitol, glycerol, meso-erythritol, and 2,3-butanediol [19]. Thus, *G. thailandicus* NBRC 3257 has unique characteristic features and the potential for the industrial production of many different oxidized products useful as drug intermediates or commodity chemicals.

**Figure 2:**
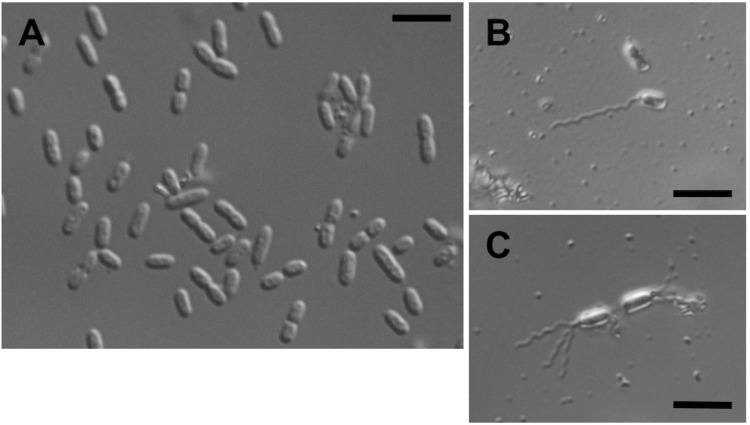
Cell morphology and flagella of *G. thailandicus* NBRC 3257. (A) Differential interference contrast image of *G. thailandicus* NBRC 3257 grown on mannitol medium. Bar, 5 µm. (B and C) Microscopic images of flagella stained by the modified Ryu method. Singly (B) and multiply (C) flagellated cells were observed. Bars, 5 µm.

*G. thanilandicus* NBRC 3257 is a strictly aerobic, mesophilic (temperature optimum ≈ 30˚C) organism. Differential interference contrast image of *G. thailandicus* NBRC 3257 cells grown on mannitol medium (25 g of D-manntiol, 5 g of yeast extract, and 3 g of polypeptone per liter) are shown in Figure 2 (A). The cells have short-rod shape with 2.6 ± 0.6 (mean ± SD, n = 10) µm in cell length and 1.2 ± 0.1 (mean ± SD, n = 10) µm in cell width. The flagella stained by the modified Ryu method are shown in Figure 2 (B) and Figure 2 (C) [20]. Singly and multiply flagellated cells were observed frequently. The characteristic features are shown in Table 1.

**Table 1:** Classification and general features of *Gluconobacter thailandicus* NBRC 3257 according to the MIGS recommendations [21]

**MIGS ID**	**Property**	**Term**	**Evidence code**^a^
	Current classification	Domain *Bacteria*	TAS [22]
		Phylum *Proteobacteria*	TAS [23]
		Class *Alphaproteobacteria*	TAS [24,25]
		Order *Rhodospirillales*	TAS [26,27]
		Family *Acetobacteraceae*	TAS [28,29]
		Genus *Gluconobacter*	TAS [27,30,31]
		Species *Gluconobacter thailandicus*	TAS [17,32]
		Strain NBRC 3257	TAS [23]
	Gram stain	Negative	IDA
	Cell shape	Rod-shaped	IDA
	Motility	Motile	NAS
	Sporulation	Not report	NAS
	Temperature range	Mesophilic	IDA
	Optimum temperature	30°C	IDA
	Carbon source	Glucose and/or glycerol	IDA
	Energy source	Glucose and/or glycerol	NAS
MIGS-6	Habitat	Free living	NAS
MIGS-6.3	Salinity	Not report	NAS
MIGS-22	Oxygen	Strict aerobes	IDA
MIGS-15	Biotic relationship	Fruits and Flower	IDA
MIGS-14	Pathogenicity	Non-pathogenic	IDA
MIGS-4	Geographic location	Japan	NAS
MIGS-5	Sample collection time	1954	NAS
MIGS-4.1	Latitude	Not report	NAS
MIGS-4.2	Longitude	Not report	NAS
MIGS-4.3	Depth	Not report	NAS
MIGS-4.4	Altitude	Not report	NAS

^a^ Evidence codes - IDA: Inferred from Direct Assay; TAS: Traceable Author Statement (i.e., a direct report exists in the literature); NAS: Non-traceable Author Statement (i.e., not directly observed for the living, isolated sample, but based on a generally accepted property for the species, or anecdotal evidence). These evidence codes are from the Gene Ontology project [33]. If the evidence code is IDA, then the property should have been directly observed, for the purpose of this specific publication, for a live isolate by one of the authors, or an expert or reputable institution mentioned in the acknowledgements.

## Genome sequencing information

### Genome project history

This genome was selected for sequencing on the basis of its phylogenetic position and 16S rRNA similarity to other members of the *Gluconobacter* genus. This Whole Genome Shotgun project has been deposited at DDBJ/EMBL/GenBank under the accession BASM00000000. The version described in this paper is the first version, BASM01000000, and the sequence consists of 107 contigs. Table 2 presents the project information and its association with MIGS version 2.0 compliance [34].

**Table 2:** Project information

**MIGS ID**	**Property**	**Term**
MIGS-31	Finishing quality	Draft
MIGS-28	Libraries used	Illumina Paired-End library
MIGS-29	Sequencing platforms	Illumina Hiseq 2000
MIGS-31.2	Fold coverage	358 ×
MIGS-30	Assemblers	Velvet ver. 1.2.07
MIGS-32	Gene calling method	Glimmer ver. 3.02
	DDBJ ID	BASM00000000
	DDBJ Date of Release	August 08, 2013
	Project relevance	Industrial

### Growth conditions and DNA isolation

The culture of strain NBRC 3257 used to prepare genomic DNA for sequencing was a laboratory stock and grown on ∆P medium [35] at 30°C with vigorous shaking. The genomic DNA was isolated as described in [36] with some modifications [35]. Three ml of culture broth was used to isolate DNA, and the final DNA preparation was dissolved in 10 mM Tris-HCl (pH 8.0) and 1 mM ethylendiamine tetraacetic acid solution. The purity, quality, and size of the genomic DNA preparation were analyzed by Hokkaido System Science Co., Ltd. (Japan) using spectrophotometer, agarose gel electrophoresis, and Qubit (Invitrogen, Carlsbad, CA) according to the their guidelines.

### Genome sequencing and assembly

The genome of *G. thailandicus* NBRC 3257 was sequenced using the Illumina Hiseq 2000 sequencing platform by the paired-end strategy (2×100 bp). Paired-end genome fragments were annealed to the flow-cell surface in a cluster station (Illumina). A total of 100 cycles of sequencing-by-synthesis were performed and high-quality sequences were retained for further analysis. The final coverage reached 358-fold for an estimated genome size of 3.44 Mb. The sequence data from Illumina HiSeq 2000 were assembled with Velvet ver. 1.2.07 [37]. The final assembly yielded 107 contigs generating a genome size of 3.44 Mb. The contigs were ordered against the complete genome of *G. oxydans* 621H [3] using Mauve [38-40].

### Genome annotation

Protein-coding genes (ORFs) of draft genome assemblies were predicted using Glimmer version 3.02 with a self-training dataset [41,42]. tRNAs and rRNAs were predicted using ARAGORN and RNAmmer, respectively [43,44]. Functional assignments of the predicted ORFs were based on a BLASTP homology search against two genome sequences, *G. thailandicus* NBRC 3255 and *G. oxydans* 621H, and the NCBI nonredundant (NR) database [45]. Functional assignment was also performed with a BLASTP homology search against Clusters of Orthologous Groups (COG) databases [46].

## Genome properties

**Figure 3:**
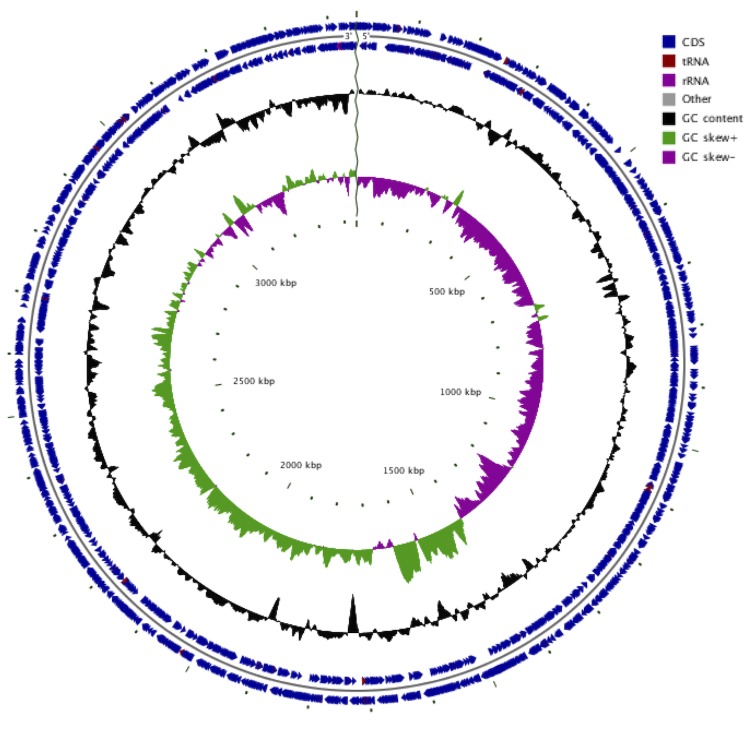
Graphical circular map of a simulated draft *Gluconobacter thailandicus* NBRC 3257 genome. The simulated genome is a set of contigs ordered against the complete genome of *G. oxydans* 621H [3] using Mauve [38-40]. The circular map was generated using CGview [47]. From the outside to the center: genes on forward strand, genes on reverse strand, GC content, GC skew.

The genome of *G. thailandicus* NBRC 3257 is 3,446,046 bp long (107 contigs) with a 56.17% G + C content (Table 3). Of the 3,414 predicted genes, 3,360 were protein coding genes, and 54 were RNAs (3 rRNA genes, and 51 tRNA genes). A total of 2,249 genes (66.93%) were assigned a putative function. The remaining genes were annotated as hypothetical genes. The properties and statistics of the genome are summarized in Table 3. The distribution of genes into COG functional categories is presented in Table 4. Of the 3,360 proteins, 2,669 (79%) were assigned to COG functional categories. Of these, 245 proteins were assigned to multiple COG categories. The most abundant COG category was "General function prediction only" (342 proteins) followed by "Amino acid transport and metabolism" (247 proteins), "Function unknown" (232 proteins), "Cell wall/membrane/envelope biogenesis" (220 proteins), "Inorganic ion transport and metabolism" (210 proteins), and "Replication, recombination and repair" (201 proteins). The genome map of *G. thailandicus* NBRC 3257 is illustrated in Figure 3, which demonstrates that the pattern of GC skew shifts from negative to positive along an ordered set of contigs with some exceptions. This suggests that the draft genome sequences were ordered almost exactly.

**Table 3:** Nucleotide content and gene count levels of the genome

**Attribute**	Value	% of total^a^
Genome size (bp)	3,446,046	-
DNA coding region (bp)	3,118,161	90.48
DNA G+C content (bp)	1,935,814	56.17
Total genes^b^	3,414	100
RNA genes	54	1.58
Protein-coding genes	3,360	98.42
Genes assigned to COGs	2,669	78.17

a) The total is based on either the size of the genome in base pairs or the total number of protein coding genes in the annotated genome.

**Table 4:** Number of genes associated with the 25 general COG functional categories

**Code**	**Value**	**%age**^a^	**Description**
J	157	4.67	Translation, ribosomal structure and biogenesis
A	0	0.00	RNA processing and modification
K	190	5.65	Transcription
L	201	5.98	Replication, recombination and repair
B	0	0.00	Chromatin structure and dynamics
D	28	0.83	Cell cycle control, cell division, chromosome partitioning
Y	0	0.00	Nuclear structure
V	44	1.31	Defense mechanisms
T	92	2.74	Signal transduction mechanisms
M	220	6.55	Cell wall/membrane/envelope biogenesis
N	43	1.28	Cell motility
Z	0	0.00	Cytoskeleton
W	2	0.06	Extracellular structures
U	95	2.83	Intracellular trafficking, secretion, and vesicular transport
O	120	3.57	Posttranslational modification, protein turnover, chaperones
C	170	5.06	Energy production and conversion
G	194	5.77	Carbohydrate transport and metabolism
E	247	7.35	Amino acid transport and metabolism
F	89	2.65	Nucleotide transport and metabolism
H	129	3.84	Coenzyme transport and metabolism
I	91	2.71	Lipid transport and metabolism
P	210	6.25	Inorganic ion transport and metabolism
Q	64	1.90	Secondary metabolites biosynthesis, transport and catabolism
R	342	10.18	General function prediction only
S	232	6.90	Function unknown
-	691	20.57	No COG assignment
-	245	7.29	Multiple COG assignment

a) The total is based on the total number of protein coding genes in the annotated genome.

### Gene repertoire of *G. thailandicus* NBRC 3257 genome

Annotation of the genome indicated that NBRC 3257 has membrane-bound PQQ-dependent alcohol dehydrogenase, *adh*AB operon (locus_tag NBRC3257_1377 and NBRC3257_1378) and *adh* subunit III (NBRC3257_1024). A unique orphan gene of *adh* subunit I was also identified (NBRC3257_3117). The gene repertories of other membrane-bound PQQ dependent proteins were investigated. Homologous proteins of membrane-bound PQQ-dependent dehydrogenase (NBRC3257_0292), membrane-bound glucose dehydrogenase (PQQ) (NBRC3257_0371), PQQ-dependent dehydrogenase 4 (NBRC3257_0662), and PQQ-dependent dehydrogenase 3 (NBRC3257_1743), were identified. In addition, two paralogous copies of the PQQ-glycerol dehydrogenase *sld*AB operon (NBRC3257_0924 to NBRC3257_0925 and NBRC3257_1134 to NBRC3257_1135) were identified.

It has been thought that the respiratory chains of *Gluconobacter* species play key roles in respiratory energy metabolism [48-51]. Therefore, the gene repertoires of respiratory chains of NBRC 3257 were also investigated. Besides two type II NADH dehydrogenase homologs (NBRC3257_1995 and NBRC3257_2785) [51], a proton-pumping NADH:ubiquinone oxidoreductase operon (type I NADH dehydrogenase complex) (NBRC3257_2617 to NBRC3257_2629), a cytochrome *o* ubiquinol oxidase *cyo*BACD operon (NBRC3257_2304 to NBRC3257_2307), and a cyanide-insensitive terminal oxidase *cio*AB operon (NBRC3257_0388 to NBRC3257_0389) [48,49], were identified.

## References

[1] Deppenmeier U Hoffmeister M Prust C Biochemistry and biotechnological applications of *Gluconobacter* strains. Appl Microbiol Biotechnol 2002; 60:233-242 10.1007/s00253-002-1114-5 12436304

[2] Sievers M Teuber M The microbiology and taxonomy of *Acetobacter europaeus* in commercial vinegar production. J Appl Microbiol 1995; 79:84-95

[3] Prust C Hoffmeister M Liesegang H Wiezer A Fricke WF Ehrenreich A Gottschalk G Deppenmeier U Complete genome sequence of the acetic acid bacterium *Gluconobacter oxydans.* Nat Biotechnol 2005; 23:195-200 10.1038/nbt1062 15665824

[4] Ge X, Zhao Y, Hou W, Zhang W, Chen W, Wang J, Zhao N, Lin J, Wang W, Chen M and others. Complete Genome Sequence of the Industrial Strain *Gluconobacter oxydans* H24. Genome Announc 2013;1(1). 10.1128/genomeA.00003-13 PMC3587919 23472221

[5] Gao L Zhou J Liu J Du G Chen J Draft genome sequence of *Gluconobacter oxydans* WSH-003, a strain that is extremely tolerant of saccharides and alditols. J Bacteriol 2012; 194:4455-4456 10.1128/JB.00837-12 22843589 PMC3416238

[6] Matsutani M Kawajiri E Yakushi T Adachi O Matsushita K Draft Genome Sequence of Dihydroxyacetone-Producing *Gluconobacter thailandicus* Strain NBRC 3255. Genome Announc 2013; 1:e0011813 23580707 10.1128/genomeA.00118-13 PMC3624681

[7] Hattori H Yakushi T Matsutani M Moonmangmee D Toyama H Adachi O Matsushita K High-temperature sorbose fermentation with thermotolerant *Gluconobacter frateurii* CHM43 and its mutant strain adapted to higher temperature. Appl Microbiol Biotechnol 2012; 95:1531-1540 10.1007/s00253-012-4005-4 22434571

[8] Sato S, Umemura M, Koike H, Habe H. Draft Genome Sequence of *Gluconobacter frateurii* NBRC 103465, a Glyceric Acid-Producing Strain. Genome Announc 2013;1(4). 10.1128/genomeA.00369-13 PMC3735062 23887908

[9] Matsutani M Hirakawa H Yakushi T Matsushita K Genome-wide phylogenetic analysis of *Gluconobacter, Acetobacter*, and *Gluconacetobacter.* FEMS Microbiol Lett 2011; 315:122-128 10.1111/j.1574-6968.2010.02180.x 21182539

[10] Matsutani M Hirakawa H Saichana N Soemphol W Yakushi T Matsushita K Genome-wide phylogenetic analysis of differences in thermotolerance among closely related *Acetobacter pasteurianus* strains. Microbiology 2012; 158:229-239 10.1099/mic.0.052134-0 22016572

[11] Peters B Mientus M Kostner D Junker A Liebl W Ehrenreich A Characterization of membrane-bound dehydrogenases from *Gluconobacter oxydans* 621H via whole-cell activity assays using multideletion strains. Appl Microbiol Biotechnol 2013; 97:6397-6412 10.1007/s00253-013-4824-y 23519735

[12] Kondo K Ameyama M Carbohydrate metabolism by *Acetobacter* species. Part I. Oxidative activity for various carbohydrates. Bull Agric Chem Soc Jpn 1958; 22:369-372 10.1271/bbb1924.22.369

[13] Takahashi M Yukphan P Yamada Y Suzuki K Sakane T Nakagawa Y Intrageneric structure of the genus *Gluconobacter* analyzed by the 16S rRNA gene and 16S-23S rRNA gene internal transcribed spacer sequences. J Gen Appl Microbiol 2006; 52:187-193 10.2323/jgam.52.187 16960335

[14] Larkin MA Blackshields G Brown NP Chenna R McGettigan PA McWilliam H Valentin F Wallace IM Wilm A Lopez R Clustal W and Clustal X version 2.0. Bioinformatics 2007; 23:2947-2948 10.1093/bioinformatics/btm404 17846036

[15] Tamura K Peterson D Peterson N Stecher G Nei M Kumar S MEGA5: molecular evolutionary genetics analysis using maximum likelihood, evolutionary distance, and maximum parsimony methods. Mol Biol Evol 2011; 28:2731-2739 10.1093/molbev/msr121 21546353 PMC3203626

[16] Tamura K Dudley J Nei M Kumar S MEGA4: Molecular Evolutionary Genetics Analysis (MEGA) software version 4.0. Mol Biol Evol 2007; 24:1596-1599 10.1093/molbev/msm092 17488738

[17] Tanasupawat S Thawai C Yukphan P Moonmangmee D Itoh T Adachi O Yamada Y *Gluconobacter thailandicus* sp. nov., an acetic acid bacterium in the alpha-*Proteobacteria.* J Gen Appl Microbiol 2004; 50:159-167 10.2323/jgam.50.159 15486825

[18] Matsushita K Nagatani Y Shinagawa E Adachi O Ameyama M Reconstitution of the ethanol oxidase respiratory chain in membranes of quinoprotein alcohol dehydrogenase-deficient *Gluconobacter suboxydans* subsp. alpha strains. J Bacteriol 1991; 173:3440-3445 1646200 10.1128/jb.173.11.3440-3445.1991 PMC207957

[19] Adachi O Fujii Y Ghaly MF Toyama H Shinagawa E Matsushita K Membrane-bound quinoprotein D-arabitol dehydrogenase of *Gluconobacter suboxydans* IFO 3257: a versatile enzyme for the oxidative fermentation of various ketoses. Biosci Biotechnol Biochem 2001; 65:2755-2762 10.1271/bbb.65.2755 11826974

[20] Kodaka H Armfield AY Lombard GL Dowell VR Jr Practical procedure for demonstrating bacterial flagella. J Clin Microbiol 1982; 16:948-952 6185531 10.1128/jcm.16.5.948-952.1982 PMC272507

[21] Field D Garrity G Gray T Morrison N Selengut J Sterk P Tatusova T Thomson N Allen MJ Angiuoli SV The minimum information about a genome sequence (MIGS) specification. Nat Biotechnol 2008; 26:541-547 10.1038/nbt1360 18464787 PMC2409278

[22] Woese CR Kandler O Wheelis ML Towards a natural system of organisms: proposal for the domains *Archaea, Bacteria*, and *Eucarya.* Proc Natl Acad Sci USA 1990; 87:4576-4579 10.1073/pnas.87.12.4576 2112744 PMC54159

[23] Garrity GM, Bell JA, Lilburn T. Phylum XIV. *Proteobacteria* phyl. nov. In: Garrity GM, Brenner DJ, Krieg NR, Staley JT (eds), Bergey's Manual of Systematic Bacteriology, Second Edition, Volume 2, Part B, Springer, New York, 2005, p. 1.

[24] Validation List No. 107. List of new names and new combinations previously effectively, but not validly, published. Int J Syst Evol Microbiol 2006; 56:1-6 10.1099/ijs.0.64188-0 16403855

[25] Garrity GM, Bell JA, Lilburn T. Class I. *Alphaproteobacteria* class. nov. In: Garrity GM, Brenner DJ, Krieg NR, Staley JT (eds), Bergey's Manual of Systematic Bacteriology, Second Edition, Volume 2, Part C, Springer, New York, 2005, p. 1.

[26] Pfennig N Truper HG Higher taxa of the phototrophic bacteria. Int J Syst Bacteriol 1971; 21:17-18 10.1099/00207713-21-1-17

[27] Skerman VBD McGowan V Sneath PHA Approved Lists of Bacterial Names. Int J Syst Bacteriol 1980; 30:225-420 10.1099/00207713-30-1-225 20806452

[28] Gillis M De Ley J Intra- and intergeneric similarities of the ribosomal ribonucleic acid cistrons of Acetobacter and Gluconobacter. Int J Syst Bacteriol 1980; 30:7-27 10.1099/00207713-30-1-7

[29] Henrici AT. The Biology of Bacteria. In: Henrici AT (ed), The Biology of Bacteria, Second Edition, Heath and Co., Chicago, 1939, p. 1-494.

[30] Asai T Taxonomic studies on acetic acid bacteria and allied oxidative bacteria isolated from fruits. A new classification of the oxidative bacteria. Nippon Nogeikagaku Kaishi 1935; 11:674-708 10.1271/nogeikagaku1924.11.8_674

[31] De Ley J, Frateur J. Genus IV. *Gluconobacter* Asai 1935, 689; emend. mut. char. Asai, Iizuka and Komagata 1964, 100. In: Buchanan RE, Gibbons NE (eds), Bergey's Manual of Determinative Bacteriology, Eighth Edition, The Williams and Wilkins Co., Baltimore, 1974, p. 251-253.

[32] Validation List no. 103. Validation of publication of new names and new combinations previously effectively published outside the IJSEM. Int J Syst Evol Microbiol 2005; 55:983-985 10.1099/ijs.0.63767-0 15879221

[33] Ashburner M Ball CA Blake JA Botstein D Butler H Cherry JM Davis AP Dolinski K Dwight SS Eppig JT Gene ontology: tool for the unification of biology. The Gene Ontology Consortium. Nat Genet 2000; 25:25-29 10.1038/75556 10802651 PMC3037419

[34] Field D Garrity G Gray T Morrison N Selengut J Sterk P Tatusova T Thomson N Allen MJ Angiuoli SV The minimum information about a genome sequence (MIGS) specification. Nat Biotechnol 2008; 26:541-547 10.1038/nbt1360 18464787 PMC2409278

[35] Kawai S Goda-Tsutsumi M Yakushi T Kano K Matsushita K Heterologous overexpression and characterization of a flavoprotein-cytochrome c complex fructose dehydrogenase of *Gluconobacter japonicus* NBRC3260. Appl Environ Microbiol 2013; 79:1654-1660 10.1128/AEM.03152-12 23275508 PMC3591945

[36] Marmur J A procedure for the isolation of deoxyribonucleic acid from microorganisms. Methods Enzymol 1963; 6:726-738 10.1016/0076-6879(63)06240-6

[37] Zerbino DR Birney E Velvet: algorithms for de novo short read assembly using de Bruijn graphs. Genome Res 2008; 18:821-829 10.1101/gr.074492.107 18349386 PMC2336801

[38] Darling AC Mau B Blattner FR Perna NT Mauve: multiple alignment of conserved genomic sequence with rearrangements. Genome Res 2004; 14:1394-1403 10.1101/gr.2289704 15231754 PMC442156

[39] Rissman AI Mau B Biehl BS Darling AE Glasner JD Perna NT Reordering contigs of draft genomes using the Mauve aligner. Bioinformatics 2009; 25:2071-2073 10.1093/bioinformatics/btp356 19515959 PMC2723005

[40] Darling AE Mau B Perna NT progressiveMauve: multiple genome alignment with gene gain, loss and rearrangement. PLoS ONE 2010; 5:e11147 10.1371/journal.pone.0011147 20593022 PMC2892488

[41] Delcher AL Bratke KA Powers EC Salzberg SL Identifying bacterial genes and endosymbiont DNA with Glimmer. Bioinformatics 2007; 23:673-679 10.1093/bioinformatics/btm009 17237039 PMC2387122

[42] Salzberg SL Delcher AL Kasif S White O Microbial gene identification using interpolated Markov models. Nucleic Acids Res 1998; 26:544-548 10.1093/nar/26.2.544 9421513 PMC147303

[43] Laslett D Canback B ARAGORN, a program to detect tRNA genes and tmRNA genes in nucleotide sequences. Nucleic Acids Res 2004; 32:11-16 10.1093/nar/gkh152 14704338 PMC373265

[44] Lagesen K Hallin P Rodland EA Staerfeldt HH Rognes T Ussery DW RNAmmer: consistent and rapid annotation of ribosomal RNA genes. Nucleic Acids Res 2007; 35:3100-3108 10.1093/nar/gkm160 17452365 PMC1888812

[45] Altschul SF Madden TL Schaffer AA Zhang J Zhang Z Miller W Lipman DJ Gapped BLAST and PSI-BLAST: a new generation of protein database search programs. Nucleic Acids Res 1997; 25:3389-3402 10.1093/nar/25.17.3389 9254694 PMC146917

[46] Tatusov RL Natale DA Garkavtsev IV Tatusova TA Shankavaram UT Rao BS Kiryutin B Galperin MY Fedorova ND Koonin EV The COG database: new developments in phylogenetic classification of proteins from complete genomes. Nucleic Acids Res 2001; 29:22-28 10.1093/nar/29.1.22 11125040 PMC29819

[47] Stothard P Wishart DS Circular genome visualization and exploration using CGView. Bioinformatics 2005; 21:537-539 10.1093/bioinformatics/bti054 15479716

[48] Mogi T Ano Y Nakatsuka T Toyama H Muroi A Miyoshi H Migita CT Ui H Shiomi K Omura S Biochemical and spectroscopic properties of cyanide-insensitive quinol oxidase from *Gluconobacter oxydans.* J Biochem 2009; 146:263-271 10.1093/jb/mvp067 19416958

[49] Miura H Mogi T Ano Y Migita CT Matsutani M Yakushi T Kita K Matsushita K Cyanide-insensitive quinol oxidase (CIO) from *Gluconobacter oxydans* is a unique terminal oxidase subfamily of cytochrome *bd.* J Biochem 2013; 153:535-545 10.1093/jb/mvt019 23526305

[50] Richhardt J Luchterhand B Bringer S Buchs J Bott M Evidence for a key role of cytochrome *bo*_3_ oxidase in respiratory energy metabolism of *Gluconobacter oxydans.* J Bacteriol 2013; 195:4210-4220 10.1128/JB.00470-13 23852873 PMC3754744

[51] Matsushita K Ohnishi T Kaback HR NADH-ubiquinone oxidoreductases of the *Escherichia coli* aerobic respiratory chain. Biochemistry 1987; 26:7732-7737 10.1021/bi00398a029 3122832

